# ZNF471 Interacts with BANP to Reduce Tumour Malignancy by Inactivating PI3K/AKT/mTOR Signalling but is Frequently Silenced by Aberrant Promoter Methylation in Renal Cell Carcinoma

**DOI:** 10.7150/ijbs.89785

**Published:** 2024-01-01

**Authors:** Xiaofei Wang, Lin Yao, Zheng Li, Jiaen Zhang, Mingjian Ruan, Yelin Mulati, Ying Gan, Qian Zhang

**Affiliations:** 1 Department of Urology, Peking University First Hospital; Institute of Urology, Peking University; Beijing Key Laboratory of Urogenital Diseases (Male) Molecular Diagnosis and Treatment Center, National Urological Cancer Center, Beijing 100034, China.; 2 Peking University Binhai Hospital, Tianjin 300450, China.

**Keywords:** ZNF471, BANP, PI3K/AKT/mTOR, methylation, renal cell carcinoma

## Abstract

**Background:** Renal cell carcinoma (RCC) is one of the most common malignant tumours of the urinary system. However, the aetiology and pathogenesis of RCC remain unclear. The C2H2 zinc finger protein (ZNF) family is the largest transcriptional regulatory factor family found in mammals, and Krüppel-associated box domain-containing zinc finger proteins (KRAB-ZFPs) constitute the largest subfamily of the C2H2 zinc finger protein family and play an important role in the occurrence and development of tumours. The aim of this study was to explore the role of abnormal methylation of ZNF471 in the development of renal carcinoma.

**Methods:** In this study, we first used the TCGA and EWAS Data Hub databases to analyse the expression and methylation levels of ZNF471 in renal carcinoma tissues and adjacent normal tissues.

Second, we collected samples of renal cancer and adjacent normal tissues at Peking University First Hospital to investigate the expression and methylation level of ZNF471 in renal cancer tissues and the relationships between these levels and the clinicopathological features and prognosis of patients with renal cancer. Next, we investigated the effects of ZNF471 on the proliferation, metastasis, cell cycle progression, and apoptosis of renal cell carcinoma cells by cell biology experiments. Finally, we elucidated the underlying molecular mechanisms of ZNF471 in renal cell carcinoma by transcriptome sequencing, bioinformatics analysis and molecular biology experiments.

**Results:** The expression of ZNF471 in renal carcinoma tissues and cell lines was significantly lower than that in adjacent normal tissues and cell lines due to abnormal promoter CpG methylation. Furthermore, the expression of ZNF471 in renal carcinoma tissues was negatively correlated with tumour stage and grade in patients with renal carcinoma. The results of the cell biology experiments showed that ZNF471 could significantly inhibit the proliferation, migration and cell cycle progression of renal cell carcinoma cells and promote apoptosis in these cells. In addition, ZNF471 could interact with BANP and suppress the malignant phenotype of RCC by inactivating the PI3K/AKT/mTOR signalling pathway.

**Conclusions:** As an important tumour suppressor, ZNF471 can interact with BANP in renal cancer cells and inhibit the activation of the PI3K/AKT/mTOR signalling pathway, thereby inhibiting the occurrence and development of renal cancer.

## Background

Renal cell carcinoma is one of the most common malignant tumours of the urinary system. According to the latest GLOBOCAN 2020 statistics released by the International Agency for Research on Cancer, approximately 430,000 new cases of RCC were diagnosed globally in 2020, accounting for 2.2% of all new cases of cancer 1-2. Partial impairment of kidney function has a compensatory capacity, thus, RCC is difficult to detect early through detection of functional abnormalities, approximately one-third of patients have metastases at diagnosis, and another one-third develop metastases during the course of the disease. RCC is a rare solid tumour that is insensitive to both radiotherapy and chemotherapy. Surgical resection is currently the most effective treatment, but up to 40% of patients still experience relapse or metastasis after resection of the primary lesion 3-4. The clinical progression of RCC is highly variable, ranging from slow growing localized tumours to tumours with highly aggressive metastatic phenotypes, and its progression and prognosis are closely related to diverse molecular events and different evolutionary patterns 5.

Epigenetics, a popular research direction in recent years, includes modifications not affecting gene sequences, such as histone modification, chromosome remodelling, regulation of noncoding RNA and DNA methylation, which ultimately affect the function and characteristics of the modified gene 6. Individual differences in drug response or prognosis can be predicted by epigenetic changes in molecular markers, and appropriate drugs, dosages and modalities can be screened to guide individualized patient treatment 7. DNA methylation refers to the covalent binding of carbon 5 on cytosine in dinucleotides of CpG islands to methyl groups under the action of a DNA methyltransferase (DNMT) 8. A large number of studies have shown that DNA methylation can bring about changes in chromatin structure and DNA conformation, thus altering DNA stability and controlling gene expression. DNA methylation is a common modification in eukaryotic cells and is also a major epigenetic form of mammalian gene expression regulation. After DNA methylation, the nucleotide sequence and composition of the gene do not change, but the expression of the gene is affected 9-12.

C2H2 zinc finger proteins(C2H2-ZNFs) originated from a small group of ancient transcription factors containing eukaryotic zinc finger structures, and after many cycles of gene replication and functional differentiation, a variety of C2H2-ZNF subfamilies eventually evolved. C2H2-ZNF family genes can be divided into 4 subfamilies based on differences in the N-terminal domain of the encoded proteins: the Finger-associated boxes (FAB) subfamily, Finger-associated repeats (FAR) subfamily, Pox virus and zinc fingers (PVZ) subfamily and Krüppel-associated box (KRAB) subfamily 13-14. KRAB-ZFPS are the largest subfamily of C2H2-ZNFs, accounting for 60% of the total C2H2-ZNF complement in humans 15. Under normal physiological conditions, regulatory factors involved in tumour occurrence and development are inactive, and some KRAB-ZNFs participate in negative regulation of these factors. In the process of tumour occurrence and further development influenced by external factors, some KRAB-ZNFs could also play a negative role in tumour occurrence and development 16-17. It has been found that members of the KRAB-ZNF family play an important role in RCC, a finding helpful for obtaining a better understanding of the molecular mechanism of RCC occurrence and searching for potential molecular markers for the diagnosis and treatment of RCC 18. As a member of the KRAB-ZNF family, ZNF471 plays an important role in the progression of various tumours. ZNF471 was found to activate the expression of MAPK10/JNK3 and PCDH family genes, and further enhance MAPK10 signaling and downstream gene expression in esophageal cancer 19. In addition, ZNF471 acted as a tumor suppressor in gastric cancer by transcriptionally inhibiting downstream targets TFAP2A and PLS3 20. ZNF471 modulated EMT and functioned as methylation regulated tumor suppressor in cervical cancer 21. However, the role of ZNF471 in renal cell carcinoma is currently unclear. Therefore, in this study, we elucidated the expression of ZNF471 and the status of promoter CpG methylation and analysed the molecular biological function and potential molecular mechanism of ZNF471 in RCC.

## Materials and Methods

### Bioinformatic analyses

The Cancer Genome Atlas (TCGA) data were analysed by using the UALCAN website (http://ualcan.path.uab.edu/. Accession date: 2022-10-20), and we obtained the expression of ZNF471 in different types of tissue samples (533 renal cancer tissues and 72 normal kidney tissues) and identified the relationships between ZNF471 expression in renal cancer tissues and clinicopathologic features (including sex, age, tumour stage, pathological grade, and lymph node metastasis status) of patients with renal cancer. In addition, we analysed the methylation level of ZNF471 in renal cancer and normal renal tissue, and identified the relationships between the methylation level of ZNF471 in renal cancer tissue and the clinicopathologic characteristics of patients with renal cancer, including sex, age, tumour stage, pathological grade, and lymph node metastasis. The GEPIA (http: //gepia.cancer-pku.cn/. Accession date: 2022-10-20) tool was used to directly generate survival curves of RCC patients with high and low ZNF471 expression levels based on an appropriate expression threshold. The Human Protein Atlas database (https://www.proteinatlas.org/. Accession date: 2022-10-30) was used to analyse the protein expression level of ZNF471 in RCC tissues and adjacent normal tissues. The EWAS Data Hub database (https://ngdc.cncb.ac.cn/ewas/datahub. Accession date: 2022-10-20) was used to analyse the relationships of the methylation level of ZNF471 and its expression in kidney tissue and the prognosis of patients with renal cancer. In this database, the methylation level of ZNF471 promoter region in 316 patients with renal cancer was detected, and they were divided into high methylation group and low methylation group according to the average methylation level of ZNF471 (0.183), where 0.183 was the average methylation level of ZNF471 promoter region in 316 patients with renal cancer. The IntAct (https://www.ebi.ac.uk/intact/home. Accession date: 2022-11-10), Pathway Commons (https://apps.pathwaycommons.org/. Accession date: 2022-11-10) and STRING (https://cn.string-db.org/. Accession date: 2022-11-10) databases were used to search for the target molecules that bind directly to ZNF471.

### Cell lines and cell culture

The human normal cell lines HK-2 and renal cancer cell lines 786-O, ACHN, Ketr-3, caki-1, caki-2, OSRC-2, 769-P and A-498 were obtained from Peking University First Hospital. These cell lines were cultured in RPMI 1640 or DMEM (Gibco BRL, Grand Island, NY) medium supplemented with 10% foetal bovine serum and 1% penicillin-streptomycin-glutamine (Gibco BRL) at 37 °C in a humidified atmosphere containing 5% CO_2_.

### Human RCC specimens

80 pairs of primary RCC tissues, and adjacent noncancerous tissues were obtained from patients who had undergone radical nephrectomy, with no prior treatment before the operation, at the Department of Urology, Peking University First Hospital. Among them, 40 were male and 40 were female. There were 43 cases aged ≤60 years old and 37 cases aged > 60 years old. Among the tumor stages, 19 cases were T1 stage, 20 cases were T2 stage, 21 cases were T3 stage and 20 cases were T4 stage. In terms of tumor size, 39 cases were ≤5cm and 41 cases were > 5cm. There were 18 cases of G1 grade, 21 cases of G2 grade, 23 cases of G3 grade and 18 cases of G4 grade (Supplementary Table 1). All collected tissue specimens were stored in liquid nitrogen immediately after surgical removal for further study. All enrolled patients signed written informed consent forms, and this study was approved by the Ethics Committee of Peking University First Hospital.

### Quantitative real‑time PCR (qRT‑PCR)

Total RNA was extracted from RCC tissues and cultured RCC cells using TRIzol reagent (Thermo Fisher Scientific) according to the manufacturer's protocols. Reverse-transcription polymerase chain reaction (RT-PCR) was carried out in a final volume of 10 µL containing 1 µg of total RNA, 7 µL of nuclease-free water, 2 µL of 5× RT Buffer, 0.5 µL of RT Enzyme Mix, and 0.5 µL of Random Primer Mix by using a ReverTra Ace qPCR RT Kit (Toyobo Co. Ltd, Osaka, Japan). Reverse transcription was performed by incubation at 37 °C for 60 minutes followed by 98 °C for 5 minutes and cooling in an ice bath for 15 seconds according to the manufacturer's instructions. Quantitative real-time PCR (qRT-PCR) was performed on an ABI 7500 (Thermo Fisher Scientific) instrument using a KAPA SYBR Green FAST qPCR Kit (KAPA, Wilmington, MA, US) according to the manufacturer's instructions. The reaction was performed in a 20 µL reaction volume containing 10 µL of KAPA SYBR® FAST qPCR Master Mix (2X) Universal, 0.4 µL of the PCR forward primer (10 µM), 0.4 µL of the PCR reverse primer (10 µM), 0.4 µL of ROX Low, 8.8 µL of double-distilled water, and less than 2 µL of template cDNA. The qRT-PCR protocol included an initial denaturation step (95 °C for 3 minutes) followed by 40 cycles of denaturation (95 °C for 15 seconds), annealing (58 °C for 30 seconds) and extension (72 °C for 1 minute). GAPDH was used as the internal control. The target gene expression levels measured by qRT‒PCR were normalized to those of GAPDH, and relative expression levels were calculated using the 2-ΔΔCT method. In this method, ∆CT is the quantitative value of the target gene (ZNF471) relative to the internal reference gene (GAPDH) in the experimental group or the control group, while 2-ΔΔCT is the fold change in the expression of the target gene (ZNF471) in the experimental group compared with that in the control group. The specific primers used for PCR are listed in Supplementary Table 2.

### DNA extraction

Genomic DNA was extracted from cell lines and tissue samples with a QIAamp DNA Mini Kit (Qiagen, Hilden, Germany). The concentrations of DNA in the samples were measured using a NanoDrop® 2000 spectrophotometer (Thermo Fisher Scientific, Waltham, MA). Sample quality was determined by gel electrophoresis, and samples were stored at -80 °C.

### 5-Aza-2′-deoxycytidine (Aza) treatment

Cells were seeded at a density of 1 × 10^5^ cells/ml in 6-well plates and grown for 24 h. Cells were then treated with 2 μM 5-Aza (Sigma-Aldrich, St Louis, MO, USA) for 96 h, with the drug and medium refreshed every day. Cells were then harvested and gene expression was analysed using RT-PCR.

### Bisulfite modification of DNA, methylation-specific PCR (MSP) and bisulfite genomic sequencing (BGS)

To evaluate the ZNF471 methylation status, bisulfite modification of DNA, MSP and BGS were performed. Genomic DNA was isolated from tissues and cell lines using a QIAamp DNA Mini kit (Qiagen, Hilden, Germany). DNA (2-5 g) was denatured in 33 ul of 0.3 mol/L NaOH at 37°C for 15 minutes, without using restriction endonuclease. Denatured DNA was mixed directly with 333 ul of bisulfite solution and treated in darkness. The bisulfite solution was prepared as either 2.4 mol/L sodium metabisulfite (pH 5.0 -5.2) /0.5mmol/L hydroquinone for a 4-hour treatment or 3.1 mol/L sodium bisulfite (pH 5.0-5.2) /0.5 mmol/L hydroquinone for a 16-hour treatment. DNA was desalted and purified using the Wizard DNA Clean-Up system. DNA was then treated with 0.3 mol/L NaOH at 37°C for 15 minutes and precipitated with 3 mol/L ammonium acetate and 3 volumes of ethanol. Recovered DNA was dissolved in 20-50 ul of TE buffer (pH 8.0) and stored at -20°C. MSP was carried out using AmpliTaqGold DNA Polymerase (Applied Biosystems) with the following thermal cycling conditions: 95 °C for 10 min followed by 40 3-step cycles (94 °C for 30 s, 58 °C for 30 s and 72 °C for 30 s), followed by a final extension step for 5 min at 72 °C. PCR products were assessed by separation on 2% agarose gels. BGS was conducted to confirm the specific amplification in bisulfite-modified DNA by BGS primers. PCR products were cloned into pEASY-T5 zero vectors (TransGen Biotech, Beijing, China), and 8 to 10 colonies were selected and sequenced randomly. The primers used for MSP and BGS were provided by Professor Qian Tao and his team from the Cancer Epigenetics Laboratory at the Chinese University of Hong Kong 19, which were listed in Supplementary Table 2.

### Small interfering RNA (siRNA)

The siRNAs against ZNF471 and BANP were obtained from GenePharma (Shanghai, China). Transfections were performed with Lipofectamine 2000 (Invitrogen), with a concentration of 10 nM siRNA in 786-O and ACHN cell lines. The cells were harvested for subsequent assays 24-48 h after transfection. The knockdown efficiency was further verified by qRT-PCR.

### Construction of plasmids and stable cell lines

The ZNF471 overexpression plasmid was purchased from Jikai Biotechnology Co., Ltd. (Shanghai, China). The expression vector of ZNF471 encoding human ZNF471 full-length open reading frame (ORF) was constructed. The DNA sequences corresponding to ZNF471 ORF were generated by RT-PCR. The sequences of PCR products were further confirmed by sequencing, and then the product was cleaved with KpnI and BamHI enzymes and ligated into pcDNA3.1(+)-Flag-V5 vector to determine the sequence and orientation. The obtained plasmid was transformed into E. coli DH5a cells and sequenced. 786-O, ACHN and caki-1 cells were plated in six-well plates and transfected with pcDNA3.1 and pcDNA3.1-ZNF471 plasmids at a concentration of 4 µg using Lipofectamine 2000 (Invitrogen, Carlsbad, CA). Forty-eight hours after transfection, puromycin was added, and the cells were incubated for 3 days to screen for cells expressing ZNF471. The efficiency of overexpression was further verified by qRT-PCR and western blotting.

### Cell viability assays

Stably transfected 786-O, ACHN and caki-1 cells were plated in 96-well plates at a density of 1000 cells/well. After the cells were adherent, Cell Counting Kit-8 (CCK-8; Dojindo Molecular Technologies, Inc., Kumamoto,Japan) reagent was added (10 μL/ per 100 μL complete medium) to assess viability at 0, 24, 48, 72, and 96 h. The absorbance at 450 nm was measured in a microplate reader.

### Colony formation assays

Stably transfected 786-O, ACHN and caki-1 cells were plated in six-well plates (800 cells/well) and cultured with various concentrations of puromycin. The stably transfected ZNF471 cells were obtained by incubation with puromycin at a constant concentration (200 μg/ml). After 10-14 d, cells were fixed with 4% PFA and stained with gentian violet (Beyotime Institute of Biotechnology, Shanghai, China). Colonies with >50 cells were photographed with a phase-contrast microscope (Leica DMI4000B, Milton Keynes, Buckinghamshire, UK), stained and then counted. Colony counts were obtained by manual counting with Photoshop software.

### Wound‑healing assay

Stably transfected 786-O, ACHN and caki-1 cells were seeded in 96-well plates, and when the confluence of the cells in the 6-well plates was greater than 90%, we used a 1-mL pipette tip to scratch a vertical artificial wound in the middle of each well. Then, the cells were washed with PBS, and new FBS-free medium was added. Images were obtained with an inverted microscope (Leica DMI4000B, Milton Keynes, Buckinghamshire, UK) at 40-X magnification. After culture for 12 and 24 h, additional cell images were obtained.

### Transwell assay of cell migration

In vitro Transwell assays were performed according to standard protocols. Transwell chambers (8-μm pore size, BD Sciences, Bedford, MA) were used to measure cell migration. The Transwell chambers were placed in 24-well plates. Cells (5×10^4^) were collected, washed twice in serum-free medium and seeded in the upper compartment. The lower compartment contained 700 μl of chemotactic medium supplemented with 10% foetal bovine serum (FBS). After incubation for 24 h, the cells were fixed with 4% paraformaldehyde for 30 min, and stained for 30 min with crystal violet. Nonmigratory cells remaining on the upper side of the filter membrane were wiped off with cotton swabs. Migrated cells were photographed with a phase-contrast microscope (Leica DMI4000B, Milton Keynes, Buckinghamshire, UK) after fixation and staining and were then counted. Five fields with evenly distributed cells were selected for counting, and the counts were averaged.

### Flow cytometric analyses of the cell cycle and apoptosis

Flow cytometry was carried out to evaluate the cell cycle and apoptosis. For cell cycle analysis, cells stably expressing ZNF471 and vector control cells were trypsinized with pancreatin, washed once with phosphate-buffered saline (PBS), and fixed with 70% ice-cold ethanol overnight. Propidium iodide (PI) was used to stain the cells for 30 min in the dark. For apoptosis analysis, cells were transfected with pcDNA3.1 and pcDNA3.1-ZNF471 plasmids at a concentration of 4 μg using Lipofectamine 2000 (Invitrogen). After 48 h, the cells were trypsinized with pancreatin, washed once with PBS, and incubated with Annexin V-fluorescein isothiocyanate (FITC; BD Pharmingen) and PI (Sigma-Aldrich) at room temperature for 30 min. The flow cytometry data were acquired using a FACSCalibur flow cytometer (BD Biosciences, Franklin Lakes, NJ, USA).

### Immunohistochemistry

Tissues previously fixed with formalin and embedded in paraffin were sliced into 4-μm sections and treated according to standard protocols, which involved the use of an anti-ZNF471 antibody (1:1000 dilution ratio) (HPA066695; Sigma-Aldrich), an anti-Ki-67 antibody (1:1000 dilution ratio) (ab15580; Abcam, Cambridge, UK) and an UltraSensitiveTM SP (Mouse/Rabbit) IHC Kit (Maxin-Bio, Fuzhou, Fujian, China) according to the manufacturer's guidance. Sections were incubated first with a primary antibody overnight at 4 °C and then with a secondary antibody (Goat Anti-Rabbit IgG H&L (HRP), 1:2000 dilution ratio) (ab205718; Abcam, Cambridge, UK) at 37 °C for 30 min. Finally, the slides were counterstained with haematoxylin. Images were captured by an inverted microscope at magnifications of 200× and 400×.

### Immunofluorescence assay

For immunofluorescence staining, cells were fixed with 4% paraformaldehyde for 20 min and then washed in PBS. The cells were permeabilized with 0.1% Triton X-100 for 20 min prior to blocking with 5% BSA for 30 min. The primary antibody (anti-ZNF471) was diluted in blocking solution and added to the cells for incubation in a humidified chamber overnight at 4 °C. A FITC-conjugated anti- rabbit secondary antibody was used. The cells were stained with DAPI (Sigma-Aldrich) for 5 min for detection of nuclei. Representative microphotographs were acquired using a Zeiss800 laser scanning confocal microscope (Zeiss).

### Western blot analysis

Total protein from tissues or cells was obtained by lysis in RIPA lysis buffer containing 1% phenylmethylsulfonyl fluoride (PMSF), and protein concentrations were measured by a bicinchoninic acid (BCA) assay kit (Beyotime Institute of Biotechnology). Equal amounts of protein (30 μg/lane) were separated by electrophoresis in 10% SDS-polyacrylamide gels, followed by transfer to a PVDF membrane (0.2 μm), blocking of the membrane with 5% nonfat milk, incubation of the membrane with primary and secondary antibodies, and detection and image acquisition with an EasySee Western blot Kit (Beijing TransGen Biotech, Beijing, China) and a chemiluminescence system (Bio-Rad, CA, USA). Primary antibodies specific for the following proteins were used: ZNF471 (1:2000 dilution ratio) (HPA066695; Sigma-Aldrich), total AKT (1:1000 dilution ratio) (sc-8312; Santa Cruz Biotechnology), phospho-AKT (1:1000 dilution ratio) (sc-7985; Santa Cruz Biotechnology), PI3K (1:1000 dilution ratio) (4257; Cell Signaling Technology), phospho-PI3K (1:1000 dilution ratio) (4228; Cell Signaling Technology), mTOR (1:1000 dilution ratio) (2983; Cell Signaling Technology), phospho-mTOR (1:1000 dilution ratio) (5536; Cell Signaling Technology), E-cadherin (1:1000 dilution ratio) (ab40772; Abcam), N-cadherin (1:5000 dilution ratio) (ab76011; Abcam), vimentin (1:1000 dilution ratio) (ab20346; Abcam), snail (1:1000 dilution ratio) (ab216347; Abcam), PARP (1:1000 dilution ratio) (9542; Cell Signaling Technology), cleaved PARP (1:1000 dilution ratio) (5625; Cell Signaling Technology), caspase 3 (1:1000 dilution ratio) (9662; Cell Signaling Technology), cleaved caspase 3 (1:1000 dilution ratio) (9661; Cell Signaling Technology), caspase 8 (1:1000 dilution ratio) (4927; Cell Signaling Technology), cleaved caspase 8 (1:1000 dilution ratio) (8592; Cell Signaling Technology), p21 (1:1000 dilution ratio) (sc-126; Santa Cruz Biotechnology), p27 (1:1000 dilution ratio) (sc-393380; Santa Cruz Biotechnology), Cyclin D1 (1:1000 dilution ratio) (1677; Epitomics, Burlingame, CA), BANP (1:10000 dilution ratio) (ab72076; Abcam), and GAPDH (1:1000 dilution ratio) (5174S; Cell Signaling Technology). The secondary antibodies were Goat Anti-Rabbit IgG H&L (HRP) (1:2000 dilution ratio) (ab205718; Abcam) and Goat Anti-Mouse IgG H&L (HRP) (1:2000 dilution ratio) (ab205719; Abcam).

### RNA sequencing (RNA-Seq)

RNA-Seq was performed by Novogene (Beijing, China). The RNA integrity number was detected with an RNA Nano 6000 Assay Kit for the Agilent Bioanalyzer 2100 system (Agilent Technologies, Santa Clara, CA). A total of 3μg RNA of per sample was used as input for the construction of cDNA libraries, whose quality was assessed on the Agilent Bioanalyzer 2100 system. After the qualified library was checked, different libraries were sequenced by illumina NovaSeg 6000 after pooling according to the requirements of effective concentration and target data volume, and the 150 bp paired end reading was generated. Image data from high-throughput sequencers were converted into sequence data (reads) by CASAVA base recognition. Then the reference genome and gene model annotation files were download from the genome website. The index of the reference genome was constructed using HISAT2 (v2.0.5), and the paired end clean reads were compared with the reference genome using HISAT2 (v2.0.5). Next, the FeatureCounts (1.5.0-p3) was used to quantify gene expression levels. Finally, DESeq2 software (1.20.0) was used to analyze the difference expression between the two comparison combinations. The methods of Benjamini and Hochberg's were used to adjust the resulting P-value (padj) to control the error discovery rate. P value<=0.05 & ïlog2 Fold Changeï>=0.0) was set as the threshold for significant differential expression. The data repository website was https://dataview.ncbi.nlm.nih.gov/?archive=bioproject and the accession number for RNA-seq data was PRJNA1035753.

### Pathway enrichment analysis

The functional enrichment of the differentially expressed genes was analysed by Novogene (Beijing, China) with GO terms and Kyoto Encyclopedia of Genes and Genomes (KEGG) pathways via the software of clusterProfiler (v 3.8.1). The level of significance was set at p<0.05.

### Coimmunoprecipitation (Co‑IP) assay

Cells were lysed in RIPA lysis buffer containing 1% PMSF and 1% protease inhibitor. A specified amount of cell lysate was retained as input, while the remaining lysate was incubated with 5 μg of a primary antibody (ZNF471, HPA066695, Sigma-Aldrich; BANP, ab72076, Abcam) or homologous IgG (Santa Cruz Biotechnology) at 4 °C overnight. Then, the lysate was incubated with 30-μl of protein A/G-beads at 4 °C for an hour, after which the beads were separated and washed with washing buffer three times. Next, proteins were isolated by elution from the beads in 2x protein loading buffer after coincubation at 100 °C for 15 min, and Western blotting was finally conducted.

### Tumor xenograft model in mice

This study was approved by the Institutional Ethics Committees of Peking University First Hospital and was conducted following the institute's guidelines. Subcutaneous xenograft mouse models were established to determine whether ZNF471 inhibits tumour growth in vivo. Fourteen male BALB/c-nude mice, 4-5 weeks old and purchased from GemPharmatech Co.,Ltd. were housed in a pathogen-free environment at the Experimental Animal Department of Peking University First Hospital. For the tumorigenesis assay, 1.0×10^7^ ACHN cells (transfected with empty vector or the ZNF471 overexpression vector) in 150 μl serum free 1640 medium containing 50% Matrigel were injected subcutaneously into the dorsal surface of each mouse, and four weeks later, the mice were euthanized, and the tumours were excised. Each group contained 7 mice. Primary tumours were measured to determine their size and weight. IHC staining was used to evaluate the expression of ZNF471 and Ki-67 in nude mouse xenografts. The anti-ZNF471 antibody was obtained from Sigma-Aldrich (HPA066695, Sigma-Aldrich), and the anti-Ki-67 antibody was obtained from Abcam (ab15580; Abcam). The sections were incubated with a primary antibody (1:100 dilution) overnight at 4 °C and then with a secondary antibody (1:2000 dilution) at 37 °C for 30 min. Finally, the slides were counterstained with haematoxylin.

Xenograft mouse models with pulmonary metastasis were established to determine whether ZNF471 inhibits tumour metastasis in vivo. Fourteen male NCG mice aged 4-5 weeks were purchased from GemPharmatech Co., Ltd. For the metastasis assay, 1.0×10^6^ 786-O cells (transfected with empty vector or the ZNF471 overexpression vector) in 150 μl pathogen-free PBS were injected into each mouse via the lateral tail vein. After 30 days, in vivo imaging was performed. Each group contained 5 mice.

### Statistical analysis

All statistical analyses were performed using SPSS 25.0 (IBM Corporation, Armonk, NY, USA) and GraphPad Prism 8.0 software (GraphPad Software Inc., La Jolla, CA, USA). The completeness of relevant information of the subjects is regarded as the quality control standard. If the information is incomplete, it will be excluded and not included in the statistics. Measurement data from at least three independent experiments are presented as the mean ± SD values. Count data are represented by [n (%)]. Student's t test and Dunnett's test were used to analyse the significance of differences between the experimental and control values. The χ2 test and Fisher's exact test were used to identify associations between the ZNF471 promoter methylation status and clinicopathological features. We used technical replicates for statistical analyses. For all analyses, p<0.05 was considered to indicate a statistically significant difference.

## Results

### The expression of ZNF471 was downregulated in renal carcinoma, and was negatively correlated with the clinical stage and pathological grade of RCC

In this study, we first used the TCGA database to analyse the expression of ZNF471 in renal cancer tissues, and the results showed that the expression of ZNF471 in most tumour tissues was significantly lower than that in adjacent normal tissues (Fig. S1A), with renal cancer tissues exhibiting this pattern (Fig. S1B). Furthermore, the expression of ZNF471 in renal carcinoma tissues was negatively correlated with clinical stage and pathological grade in patients with renal carcinoma but was not correlated with patient sex or age (Fig. S1C-G). In addition, the patients with high expression of ZNF471 had a higher overall survival rate than those with low expression (Fig. S1H). Using The Human Protein Atlas database, we found that the protein expression level of ZNF471 in renal cancer tissues was lower than that in adjacent normal tissues (Fig. S1I-J).

**Figure 1:**
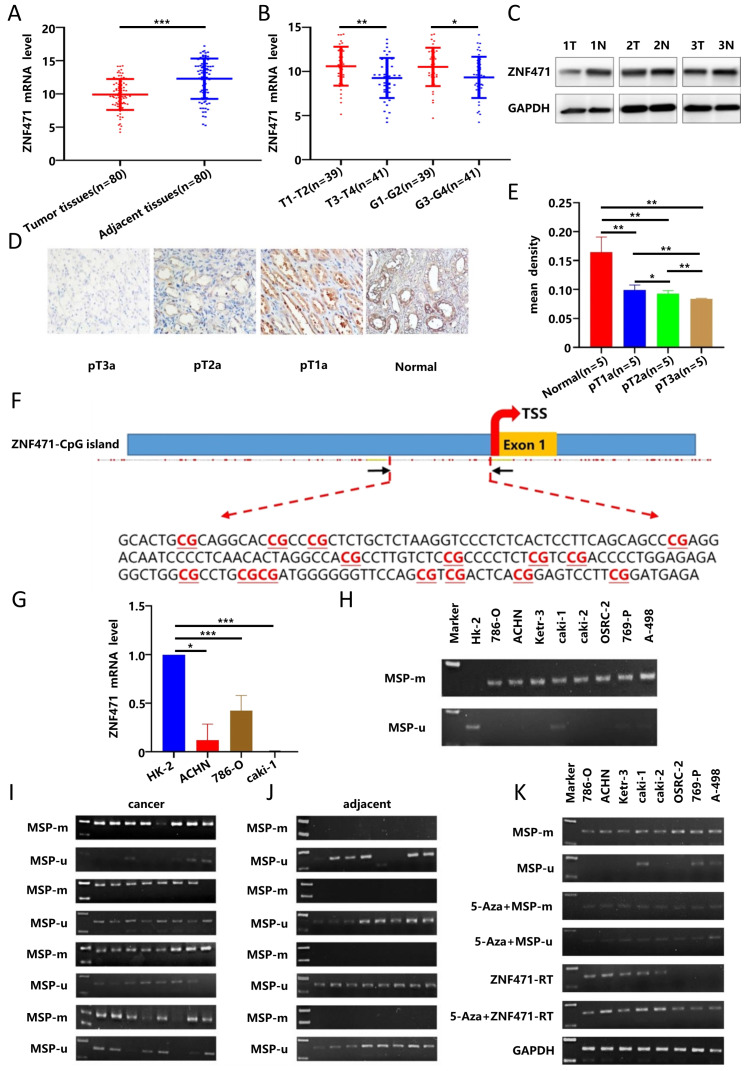
The low expression of ZNF471 in renal carcinoma was due to promoter CpG hypermethylation. A. The mRNA expression level of ZNF471 in RCC tissues and adjacent normal tissues. B. The correlations between the expression of ZNF471 in RCC tissues and clinical stage and pathological grade in patients with RCC. C-E. The protein expression level of ZNF471 in renal cancer tissues (T: Renal cancer tissue, N: Adjacent normal tissue). F. A typical CpG island in the ZNF471 promoter. G. The expression of ZNF471 in renal cancer cell lines and normal cell line. H. The methylation level of ZNF471 in renal cancer cell lines and a normal cell line (HK-2 is normal cell line and 786-O, ACHN, Ketr-3, caki-1, caki-2, OSRC-2, 769-P and A-498 are renal cancer cell lines). I-J. The methylation level of ZNF471 in RCC tissues and adjacent normal tissues. K. The expression and methylation status of ZNF471 upon 5-aza-2-deoxycytidine (Aza) treatment in RCC cell lines. (n.s: p>0.05, *p<0.05, **p<0.01, ***p<0.001).

Subsequently, 80 pairs of RCC tissues and adjacent normal tissues were collected in our hospital for verification, and the results showed that the expression of ZNF471 in RCC tissues was significantly lower than that in adjacent normal tissues (Fig. 1A), and that there were significant negative correlations between the expression of ZNF471 in RCC tissues and clinical stage and pathological grade in these patients with RCC(Fig. 1B, Table 1), consistent with the TCGA database analysis results. By western blot analysis, we found that the expression of ZNF471 in adjacent normal tissues was higher than that in renal cancer tissues (Fig. 1C). In addition, by immunohistochemical analysis, we found that the expression of ZNF471 in normal kidney tissues was higher than that in renal cancer tissues and that the expression of ZNF471 decreased with increasing postoperative pathological stage of the tumour (Fig. 1D-E).

### ZNF471 was downregulated by CpG methylation in RCC

We first used data from the TCGA database to analyse the methylation of ZNF471 in RCC, and the results showed that the methylation level of ZNF471 in renal carcinoma tissues was significantly higher than that in adjacent normal tissues (Fig. S2A). In addition, the results of TCGA database showed that the methylation level of ZNF471 in renal cancer tissues was positively correlated with high clinical stage, lymph node metastasis, and high pathological grade in patients with RCC, but not with patient sex or age (Fig. S2B-F). In our study, we found that the methylation level of ZNF471 in renal cancer tissues was positively correlated with high clinical stage, but not with pathological grade, tumor size, sex and age in patients with RCC (Table 2). Through analysis of the EWAS Data Hub database, we found that the overall survival rate of RCC patients with hypermethylation was lower than that of patients with hypomethylation (Fig. S2G) and that there was a significant negative correlation between the expression level and the methylation level of ZNF471 in RCC tissues (Fig. S2H).

Sequence analysis by CpG Island Searcher confirmed that the ZNF471 promoter contains a CpG island (Fig. 1F), and indicated that CpG methylation may regulate its expression by this mechanism. Subsequently, we used RT-PCR and methylation-specific PCR to quantify the expression and methylation levels of ZNF471 in renal cancer cell lines and found that the expression of ZNF471 in renal cancer cell lines was lower than that in normal cell lines (Fig. 1G). The methylation level of ZNF471 in the renal cancer cell line was higher than that in the normal cell line (Fig. 1H). Furthermore, we also verified this finding in RCC tissues, with similar results (Fig. 1I-J). Highresolution bisulfite genomic sequencing (BGS) analysis was conducted to examine the methylation status of 43 individual CpG sites within the ZNF471 promoter, with a higher density of methylated alleles were observed in RCC cell lines (786-O and ACHN) and RCC tissues compared with normal cell lines (HK-2) and adjacent normal tissues, consistent with the MSP results (Fig. S2I-J).

To further verify whether the low expression of ZNF471 in renal cancer tissues is related to the high promoter CpG methylation, we treated renal cancer cell lines with 5-Aza, DNA methyltransferase inhibitor, and the results showed that in renal cancer cell lines, the methylation level of ZNF471 was decreased, while the expression level of ZNF471 was increased (Fig. 1K). In conclusion, these results suggested that the low expression of ZNF471 in renal carcinoma might be due to promoter CpG hypermethylation.

### ZNF471 inhibited the proliferation of renal cancer cells

**Figure 2:**
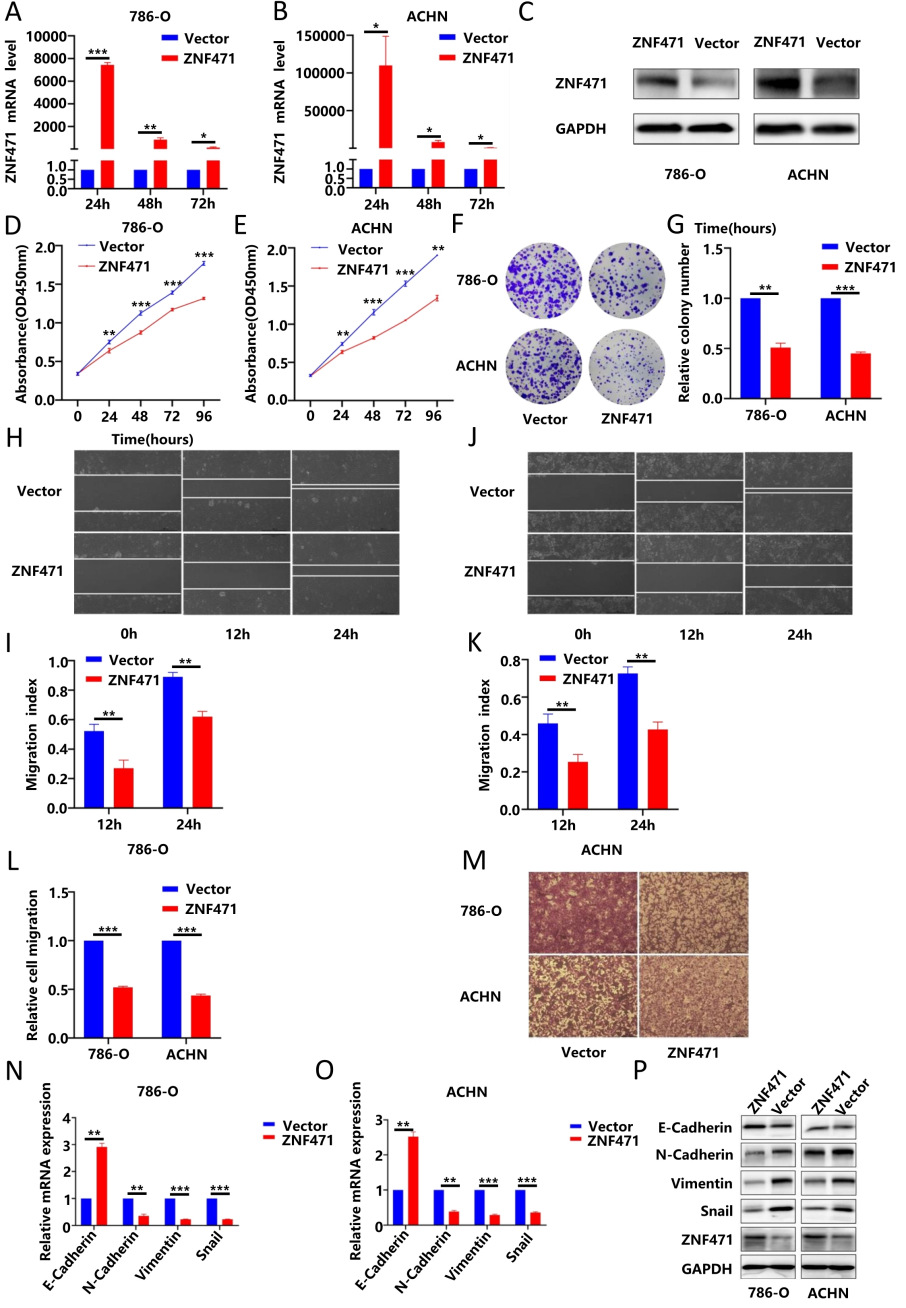
ZNF471 inhibited the proliferation and invasion of RCC cells. A-C. The ZNF471 overexpression efficiency in RCC cell lines. D-E. The effect of ZNF471 on proliferation in RCC cell lines, as determined by a CCK-8 assay. F-G. The effect of ZNF471 on proliferation in RCC cell lines, as determined by a plate colony formation assay. H-K. The effect of ZNF471 on invasion in RCC cell lines, as determined by a wound healing assay. L-M. The effect of ZNF471 on invasion in RCC cell lines, as determined by a Transwell assay. N-P. The effect of ZNF471 on the mRNA and protein levels of E-cadherin, N-cadherin, vimentin and snail in RCC cell lines. (n.s: p>0.05, *p<0.05, **p<0.01, ***p<0.001).

By using immunofluorescence techniques, we found that ZNF471 was localized primarily in the nucleus in renal cancer cells (Fig. S3A). To verify the biological function of ZNF471 in renal cancer, we transfected the pcDNA3.1-ZNF471 expressing plasmid into renal cancer cell lines (786-O, ACHN, and caki-1), and then, the ZNF471 overexpression efficiency was analysed by qRT-PCR after transfection for 24 h, 48 h and 72 h. We found that compared with the control group, the ZNF471 overexpression group exhibited the highest overexpression efficiency after transfection for 24 h (Fig. 2A-B, Fig. S3B). In addition, the results of western blot showed that the protein level in the ZNF471 overexpression group was significantly higher than that in the Vector group (Fig. 2C, Fig. S3C). Therefore, we selected renal cancer cell lines transfected for 24 h for follow-up experiments. Through CCK-8 assays and plate colony formation assays, we found that the proliferation ability of renal cancer cells in the ZNF471 overexpression group was significantly lower than that of renal cancer cells in the control group (Fig. 2C-G, Fig. S3D-F).

### ZNF471 could inhibit the invasion of renal cancer cells by regulating EMT

Invasion and metastasis play an important role in tumour progression. Therefore, to investigate the effect of ZNF471 on the invasion and metastasis of renal carcinoma cells, wound healing assays and Transwell migration assays were performed. The results showed that the invasion and metastasis abilities of renal cancer cells in the ZNF471 overexpression group were significantly lower than those of renal cancer cells in the control group (Fig. 2H-M, Fig. S3G-J). EMT plays an important role in tumour metastasis. Therefore, we analysed the effect of ZNF471 on EMT by qRT-PCR and Western blot analyses. The results showed that compared with those in the control group, the mRNA and protein levels of E-cadherin in the ZNF471 overexpression group were increased, while the mRNA and protein levels of N-cadherin, vimentin and snail were decreased (Fig. 2N-P, Fig. S4A-B). These results suggested that ZNF471 could inhibit the metastasis of renal cancer cells by regulating EMT.

### ZNF471 induced apoptosis and cell cycle arrest in renal carcinoma cells

**Figure 3:**
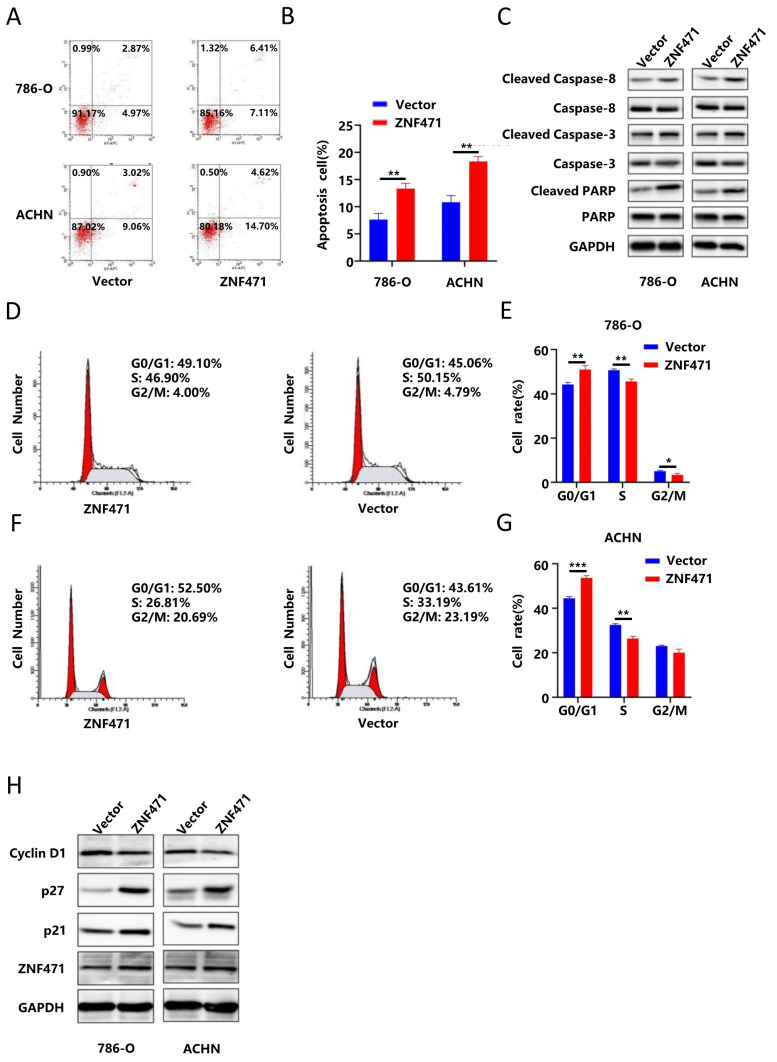
ZNF471 induced apoptosis and cell cycle arrest in RCC cell lines. A-B. The effect of ZNF471 on apoptosis in RCC cell lines. C. The effect of ZNF471 on the protein levels of caspase 3, cleaved caspase 3, caspase 8, cleaved caspase 8, PARP and cleaved PARP in RCC cell lines. D-G. The effect of ZNF471 on the cell cycle in RCC cell lines. H. The effect of ZNF471 on the protein levels of p21, p27 and Cyclin D1 in RCC cell lines. (n.s: p>0.05, *p<0.05, **p<0.01, ***p<0.001).

To study the effect of ZNF471 on apoptosis in renal cancer cells, flow cytometry was performed, and the results showed that the number of apoptotic renal cancer cells in the ZNF471 overexpression group was significantly higher than that in the control group (Fig. 3A-B, Fig. S3K-L). In addition, apoptosis can be activated through the caspase cascade.

Therefore, we measured the protein levels of caspase 3, cleaved caspase 3, caspase 8, cleaved caspase 8, PARP and cleaved PARP by Western blotting. The results showed that compared with those in the control group, the protein levels of cleaved caspase 3, cleaved caspase 8 and cleaved PARP were significantly increased in the ZNF471 overexpression group (Fig. 3C, Fig. S4C-D). To further explore whether ZNF471 can inhibit the proliferation of renal cancer cells by affecting cell cycle progression, we conducted cell cycle analysis, and the results showed that compared with that in the control group, the number of G0/G1 phase cells was significantly increased but the numbers of S phase and G2/M phase cells were significantly decreased in the ZNF471 overexpression group (Fig. 3D-G, Fig. S3M-N). In addition, Western blotting was used to measure the expression of the cell cycle-related proteins p21, p27 and cyclin D1. The results showed that in the ZNF471 overexpression group, the expression of p21 and p27 was significantly higher than that in the control group, while the expression of cyclin D1 was significantly lower than that in the control group (Fig. 3H, Fig. S4E-F).

### Knockdown of ZNF471 promoted the growth of renal cancer cells and induced their metastasis

**Figure 4:**
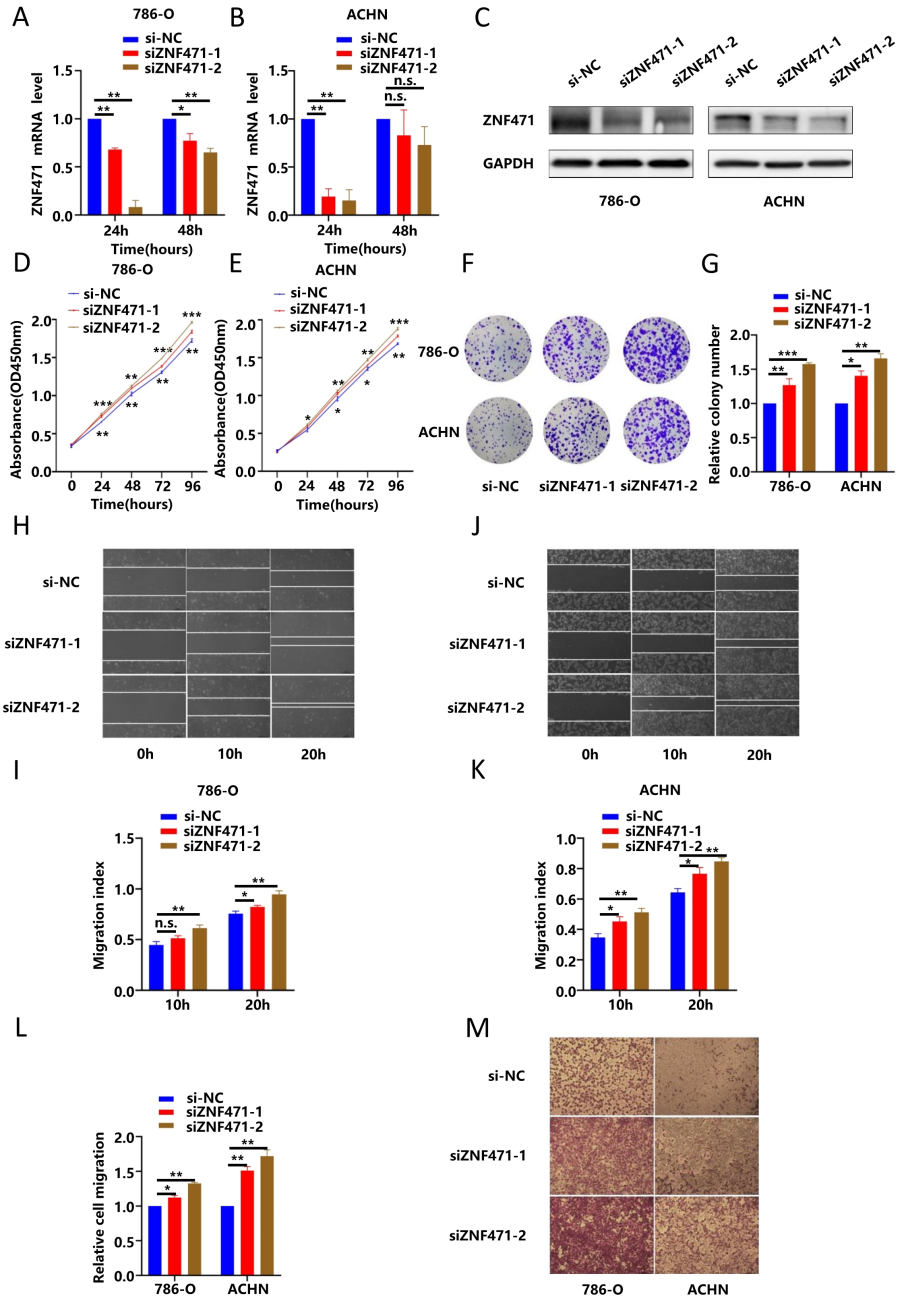
ZNF471 knockdown promoted the growth of renal cancer cells and induced their metastasis. A-C. The ZNF471 knockdown efficiency in RCC cell lines. D-E. The effect on proliferation after knockdown of ZNF471 in RCC cell lines, as determined by a CCK-8 assay. F-G. The effect on proliferation after knockdown of ZNF471 in RCC cell lines, as determined by a plate colony formation assay. H-K. The effect on invasion after knockdown of ZNF471 in RCC cell lines, as determined by a wound healing assay. L-M. The effect on invasion after knockdown of ZNF471 in RCC cell lines, as determined by a Transwell assay. (n.s: p>0.05, *p<0.05, **p<0.01, ***p<0.001).

To further verify the inhibitory effects of ZNF471 on renal cancer cells, we used a siRNA targeting ZNF471 to knock down the expression of ZNF471 in renal cancer cell lines, and then we used qRT-PCR to evaluate the knockdown efficiency after transfection for 24 h and 48 h. The results showed that compared with the control group, the ZNF471 knockdown group of renal cancer cell lines exhibited the highest knockdown efficiency after transfection with siRNA for 24 h (Fig. 4A-B). In addition, the results of western blot showed that the protein level in the ZNF471 knockdown group was significantly lower than that in the control group (Fig. 4C). Therefore, we selected renal cancer cells transfected for 24 h for the next experiments. Through CCK-8 assays and plate colony formation assays, we found that the proliferation ability of renal cancer cells in the ZNF471 knockdown group was significantly higher than that of renal cancer cells in the control group (Fig. 4D-G). In addition, the results of the wound healing assay and Transwell migration assay showed that the invasion and metastasis abilities of renal cancer cells in the ZNF471 knockdown group were significantly increased compared with those of renal cancer cells in the control group (Fig. 4H-M).

### ZNF471 inhibited tumour growth and metastasis in vivo

**Figure 5:**
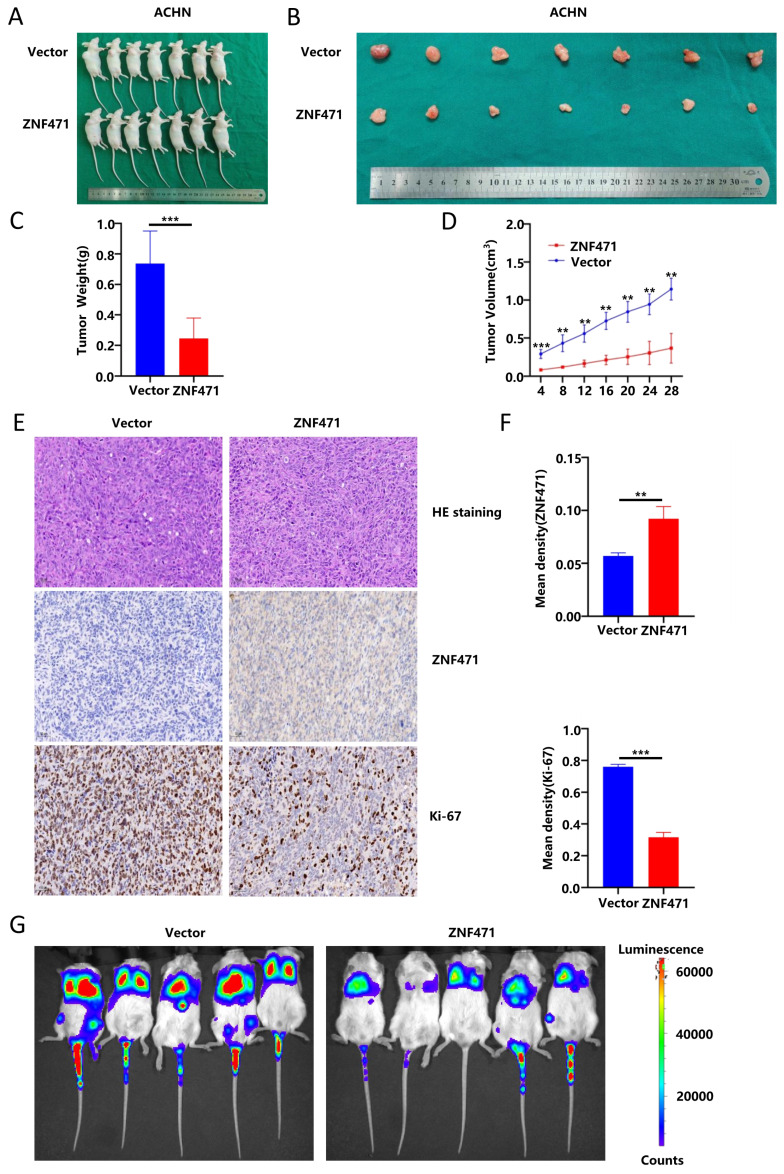
ZNF471 inhibited tumour growth and metastasis in vivo. A-D. The volume and weight of tumours in nude mice in the ZNF471 overexpressing group and control group. E-F. Representative photographs of H&E staining and IHC staining of ZNF471 and Ki-67. G. The fluorescence intensity in the lungs of NCG mice injected with cells with ZNF471 overexpression and control cells. (n.s: p>0.05, *p<0.05, **p<0.01, ***p<0.001).

To further investigate the biological functions of ZNF471 in vivo, a nude mouse xenograft model was used. ACHN cells stably transfected with ZNF471 were subcutaneously injected into BALB/c nude mice while ACHN cells transfected with empty vector were used similarly as the negative control cells. Four weeks after injection, the volume and weight of tumours in nude mice were significantly smaller in the ZNF471-overexpressing group than in the control group (Fig. 5A-D). Moreover, the histological features of the xenografts in the nude mice were analysed by haematoxylin and eosin (HE) staining, and the expression levels of ZNF471 and Ki-67 were analysed by immunohistochemical (IHC) staining. The results of HE and IHC staining showed that the number of tumour cells with robust nuclear fragmentation was increased in xenografts with ZNF471 overexpression, accompanied by a decrease in Ki-67 staining (Fig. 5E-F). Next, to further test whether ZNF471 can inhibit renal cancer metastasis in vivo, ZNF471-overexpressing and empty vector-expressing ACHN cells were injected into the tail vein of NCG mice, and lung colonization was analysed 30 days later. The results showed that the fluorescence intensity in the lungs of NCG mice injected with ZNF471-overexpressing cells was significantly weaker than that in the lungs of control group mice (Fig. 5G).

### ZNF471 could exert tumour suppressive effects by inhibiting the PI3K/AKT/mTOR signalling pathway

**Figure 6:**
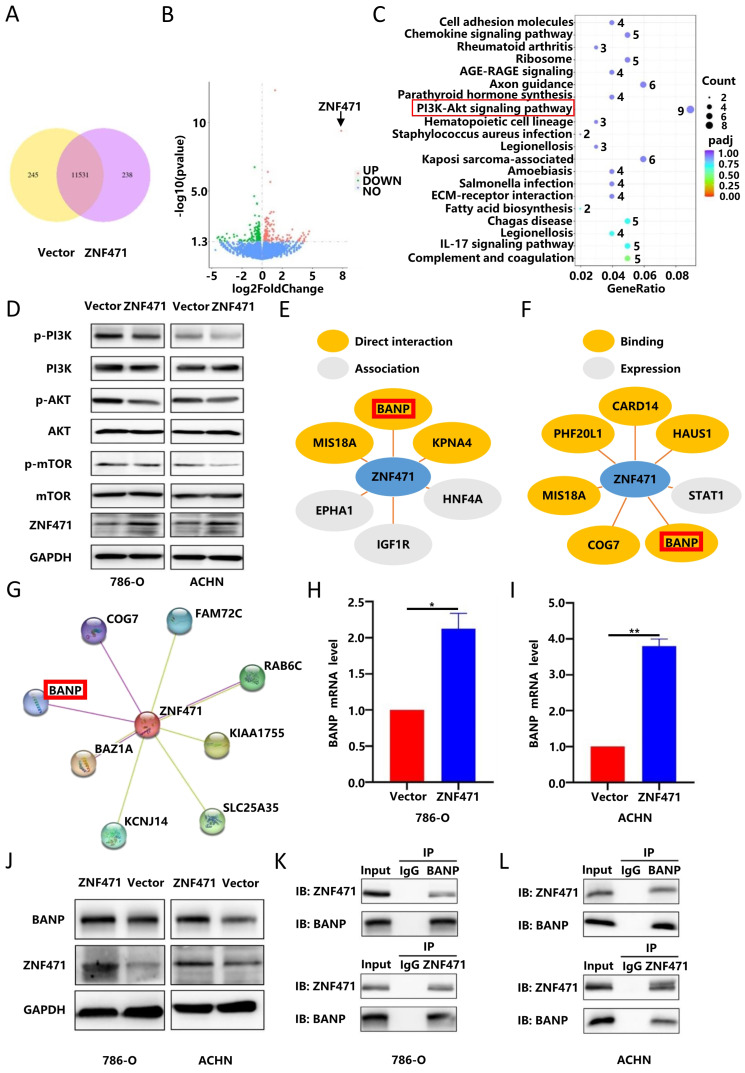
ZNF471 could interact with BANP to inhibit the activation of the PI3K/AKT/mTOR signalling pathway. A-B. Transcriptome sequencing in renal cancer cell lines overexpressing ZNF471. C. KEGG functional enrichment analyses of the differentially expressed genes. D. The anticancer effects of ZNF471 through inhibition of PI3K/AKT/mTOR activation. E. Proteins with direct interactions with ZNF471, including MIS18A, BANP and KPNA4, based on IntAct analysis. F. The proteins binding to ZNF471, including MIS18A, COG7, PHF20L1, CARD14, BANP and HAUS1, identified in the Pathway Commons database. G. The interaction between ZNF471 and BANP, as determined through STRING database analysis. H-J. The correlation between the expression of ZNF471 and BANP, as determined by qRT-PCR and Western blot analyses. K-L. ZNF471 could physically bind to BANP, as determined by a coimmunoprecipitation assay. (n.s: p>0.05, *p<0.05, **p<0.01, ***p<0.001).

To further explore the molecular mechanism underlying the antitumour effect of ZNF471, transcriptome sequencing was performed on renal cancer cells overexpressing ZNF471 to search for differentially expressed genes, and pathway enrichment analysis was also performed. The research results are shown in Fig. 6A-B. The results of KEGG functional enrichment analyses suggested that ZNF471 could play a role through the PI3K/AKT signalling pathway (Fig. 6C). In addition, studies have found that the PI3K/AKT/mTOR signalling pathway is closely related to the malignant phenotype of renal cancer and plays an important role in the occurrence and development of renal cancer. Therefore, we speculated that ZNF471 might play an antitumour role by regulating PI3K/AKT/mTOR signalling. Based on this hypothesis, the expression levels of key proteins of the PI3K/AKT/mTOR pathways were measured by Western blotting. The results showed that compared with those in the control group, the protein levels of phospho-PI3K, phospho-AKT and phospho-mTOR in the ZNF471 overexpression group were significantly decreased (Fig. 6D, Fig. S4G-H). Therefore, ZNF471 could exert anticancer effects by inhibiting PI3K/AKT/mTOR pathway activation.

### ZNF471 could bind to BANP and promote BANP expression

In addition, ZNF471 has been confirmed be able to participate in protein binding and protein modification. Based on analysis of data in IntAct, a protein-protein interaction database, we found a number of proteins that directly interact with ZNF471, including MIS18A, BANP and KPNA4(Fig. 6E). In addition, by analysing the Pathway Commons database, we found a number of proteins binding to ZNF471, including MIS18A, COG7, PHF20L1, CARD14, BANP and HAUS1 (Fig. 6F). In addition, we found an interaction between ZNF471 and BANP through STRING database analysis (Fig. 6G). Based on the results of the above three database analyses, we selected BANP as a target interaction partner of ZNF471 to investigate because of its critical role in tumorigenesis and development. Moreover, we found a positive correlation between the expression of ZNF471 and that of BANP through qRT-PCR and Western blot analyses (Fig. 6H-J, Fig. S4I-J). Furthermore, by a coimmunoprecipitation assay, we found that ZNF471 can physically bind to BANP (Fig. 6K-L). In conclusion, ZNF471 could physically interact with BANP and positively regulate BANP protein expression in RCC.

### BANP could inhibit the proliferation and metastasis of renal carcinoma cells

**Figure 7:**
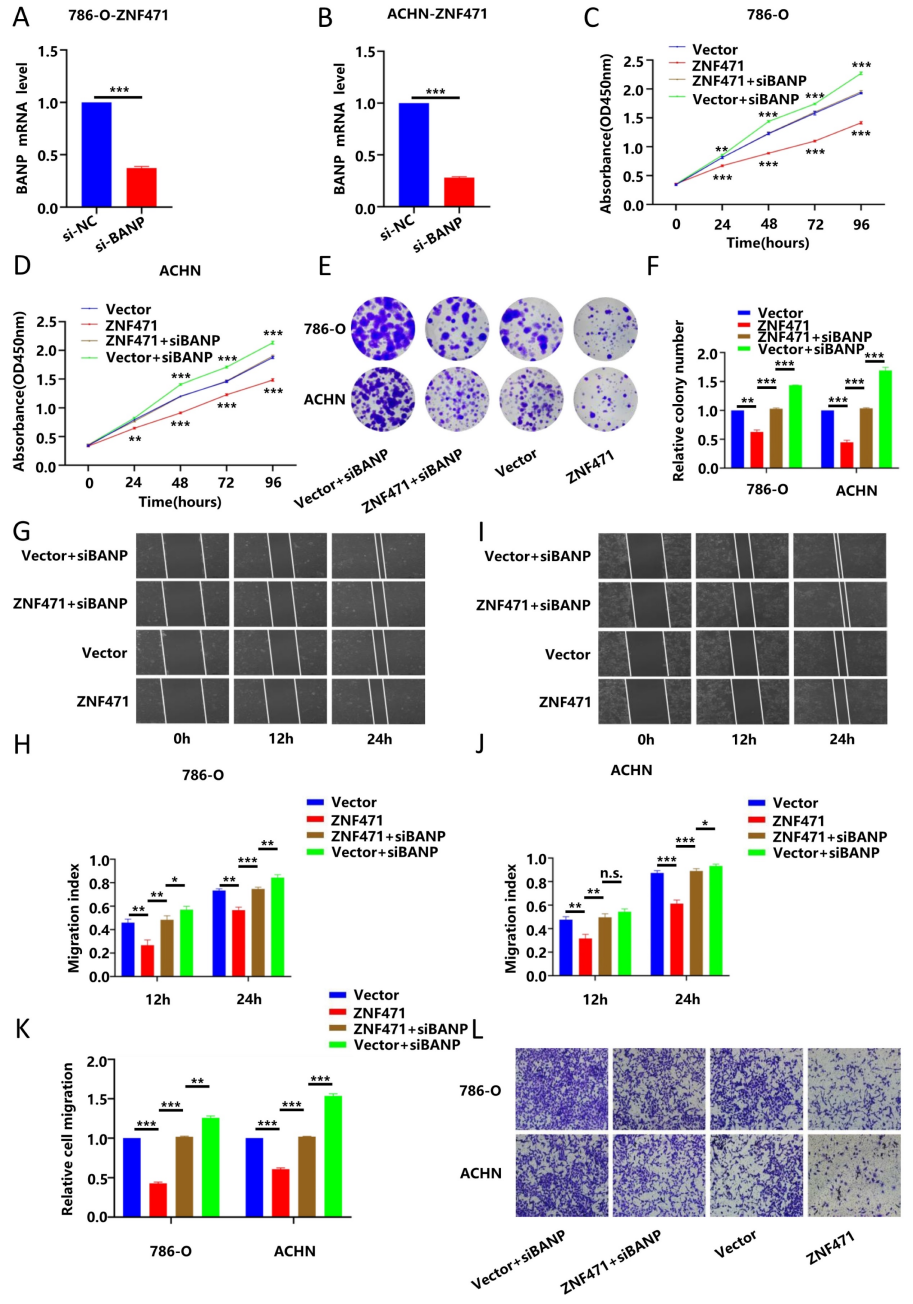
BANP inhibited the proliferation and metastasis of renal carcinoma cells. A-B. The knockdown efficiency of BANP in renal cancer cell lines stably transfected with ZNF471. C-D. The ability of proliferation in four groups of Vector, ZNF471, ZNF471+siBANP and Vector+siBANP, as determined by a CCK-8 assay. E-F. The ability of proliferation in four groups of Vector, ZNF471, ZNF471+siBANP and Vector+siBANP, as determined by a plate colony formation assay. G-J. The ability of invasion in four groups of Vector, ZNF471, ZNF471+siBANP and Vector+siBANP, as determined by a wound healing assay. K-L. The ability of invasion in four groups of Vector, ZNF471, ZNF471+siBANP and Vector+siBANP, as determined by a Transwell assay. (n.s: p>0.05, *p<0.05, **p<0.01, ***p<0.001).

To verify the biological function of BANP in renal cancer, we transfected a BANP-specific siRNA into renal cancer cells (786-O and ACHN) stably transfected with ZNF471 to knock down the expression of BANP, and then qRT-PCR was used to determine the knockdown efficiency after transfection (Fig. 7A-B). Then, we divided the study into four groups, namely Vector, ZNF471, ZNF471+siBANP and Vector+siBANP. Through CCK-8 assays and plate colony formation assays, we found that compared with Vector group, the proliferation ability was significantly inhibited in ZNF471 group. Compared with the ZNF471 group, the proliferation ability in ZNF471+siBANP group was enhanced. In addition, compared with Vector+siBANP group, the proliferation ability in ZNF471+siBANP group was significantly inhibited (Fig. 7C-F). Through wound healing assays and Transwell migration assays, we found that compared with Vector group, the invasion and metastasis abilities were significantly inhibited in ZNF471 group. Compared with the ZNF471 group, the invasion and metastasis abilities in ZNF471+siBANP group were enhanced. Furthermore, compared with Vector+siBANP group, the invasion and metastasis abilities in ZNF471+siBANP group were significantly inhibited (Fig. 7G-L).

### BANP could induce apoptosis and cell cycle arrest in renal carcinoma cells

**Figure 8:**
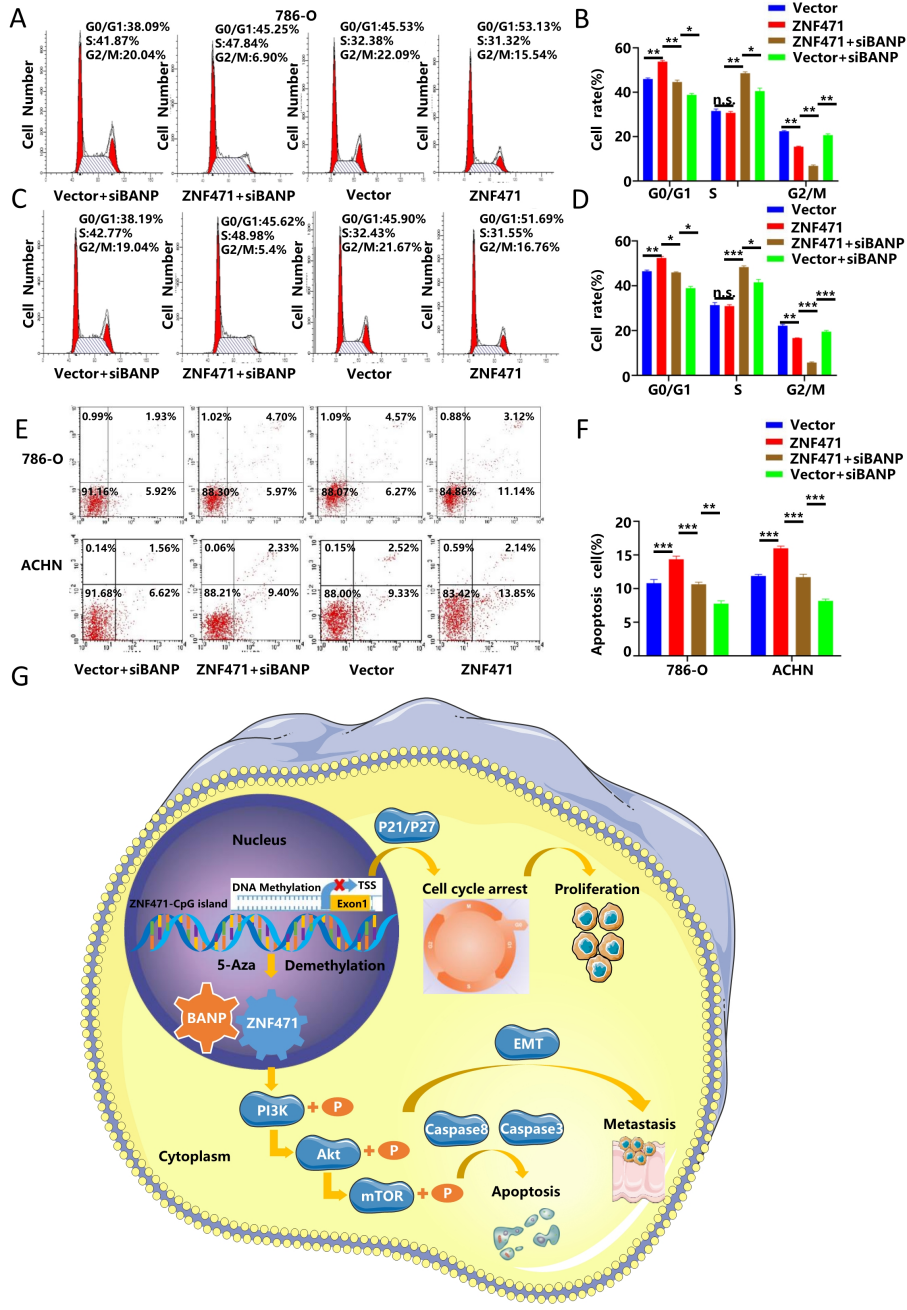
BANP induced apoptosis and cell cycle arrest in renal carcinoma cells. A-D. The comparison of cell cycle in four groups of Vector, ZNF471, ZNF471+siBANP and Vector+siBANP. E-F. The comparison of cell apoptosis in four groups of Vector, ZNF471, ZNF471+siBANP and Vector+siBANP. G. A molecular model showing the possible mechanism underlying ZNF471-mediated tumour suppression in RCC. (n.s: p>0.05, *p<0.05, **p<0.01, ***p<0.001).

To further explore whether BANP could inhibit the proliferation of renal cancer cells by affecting the cell cycle, we conducted cell cycle analysis, and the results showed that compared with Vector group, the progression of cell cycle was significantly inhibited in ZNF471 group. Compared with the ZNF471 group, the progression of cell cycle in ZNF471+siBANP group was promoted. In addition, compared with Vector+siBANP group, the progression of cell cycle in ZNF471+siBANP group was significantly inhibited (Fig. 8A-D). To investigate the effect of BANP on apoptosis in renal cancer cells, a flow cytometry assay was performed, and the results showed that compared with Vector group, the number of apoptotic renal cancer cells was significantly increased in ZNF471 group. Compared with the ZNF471 group, the number of apoptotic renal cancer cells in ZNF471+siBANP group was reduced. In addition, compared with Vector+siBANP group, the number of apoptotic renal cancer cells in ZNF471+siBANP group was significantly increased (Fig. 8E-F). In summary, these results suggested that ZNF471 could interact with BANP in renal cancer cells and inactivate PI3K/AKT/mTOR signalling, thus further suppressing the malignant phenotype of RCC cells (Fig. 8G).

## Discussion

During mammalian evolution, most transcription factor families were structurally and functionally conserved, but the KRAB-ZNF subfamily, which is currently the largest transcriptional regulatory factor family in mammals, has acquired species specificity 22. The classical KRAB-ZFP structure consists of two parts, namely, the N-terminal KRAB domain and the C-terminal tandem repeat zinc finger domain (ZNF). The KRAB domain mainly interacts with KAP1 in the TRIM family (tripartite motif family) to jointly inhibit gene transcription, while the zinc finger domain mainly mediates the binding of zinc finger proteins to DNA sequences 23-24. ZNF471, a member of the KRAB C2H2-type ZFP family, was found to be silenced by promoter hypermethylation in gastric, colon, tongue squamous, oesophageal, and breast cancers 19-20, 25-27. However, the function and mechanism of ZNF471 in renal cell carcinoma remain unclear. In this study, we found that the expression of ZNF471 in renal cancer tissues and cell lines was significantly lower than that in the corresponding paracancerous tissues and in normal cell lines and revealed that the low expression of ZNF471 in renal cancer tissues and cell lines was closely related to hypermethylation of the promoter region.

Studies have shown that KRAB-ZFPs have important biological functions in a variety of tumours, that include modulation of cell proliferation, metastasis, and apoptosis 28. In this study, we found that overexpression of ZNF471 significantly inhibited the proliferation and metastasis of renal cancer cells, and induced apoptosis and cell cycle arrest in renal cancer cells. The expression of apoptotic markers was analysed to confirm its proapoptotic effect. In the ZNF471 overexpression group, the protein levels of cleaved caspase 3, cleaved caspase 8 and cleaved PARP were significantly increased. Analysis of cell cycle-related protein expression indicated that the expression of p21 and p27 in the ZNF471 overexpression group was significantly higher than that in the control group, while the expression of Cyclin D1 was significantly lower than that in the control group. In addition, ZNF471 reversed EMT to inhibit the invasion of renal cancer cells. In contrast, knocking down ZNF471 promoted the growth of renal cancer cells and induced their metastasis. ZNF471 inhibited tumour growth and metastasis in vivo.

Studies have shown that the activation of the PI3K/AKT/mTOR signalling pathway, which can accelerate the cell cycle, inhibit apoptosis, and promote tumour cell migration is closely related to the occurrence of tumours 29. In addition, cyclin, cyclin-dependent kinase (CDK) and cyclin-dependent kinase inhibitor (CKI) proteins regulate the cell cycle, and activated AKT can promote the degradation of cyclin D1 and accelerate the G1-S transition, thus promoting cell proliferation 30-31. In addition, AKT was found to directly inhibit p21 after activation, abolishing the inhibitory effect of p21 on the formation of the cyclin kinase cyclinD1-CDK4-CDK6 complex, inactivate the phosphorylation of the retinoblastoma suppressor protein (pRb) and finally promote the G1-S transition 32. Apoptosis is a normal cellular function that controls excessive cell proliferation. Activation of the PI3K/AKT/mTOR signalling pathway can inhibit apoptosis and promote cell survival. Bad, a member of the Bcl-2 family, can form a complex with Bcl-2 or Bcl-xL to accelerate apoptosis. Activated AKT can phosphorylate Serl36 of Bad, thereby inhibiting the dimerization of Bad with Bcl-2 or Bcl-xL and thus suppressing the proapoptotic effect of Bad 33-35. In this study, we explored the potential molecular mechanism of ZNF471's inhibitory role in renal cancer by transcriptome sequencing. The protein levels of phospho-PI3K, phospho-AKT, and phospho-mTOR in the ZNF471 overexpression group were significantly lower than those in the control group. Therefore, ZNF471 could suppress the malignant phenotype of renal cancer by inhibiting the activation of the PI3K/AKT/mTOR signalling pathway.

BANP (also known as SMAR1) is encoded by a gene located on chromosome 16q24.3 in humans and is expressed in heart, liver, spleen, lung, kidney, brain and thymus tissues, with the highest expression in the thymus 36. The main role of BANP in tumours is to regulate the cell cycle, prevent tumour metastasis and invasion through the transforming growth factor (TGF-β) pathway, and regulate cytoskeletal dynamics and the NF-κB signalling pathway 37. Studies have found that BANP could arrest the cell cycle at the G1/S and G2/M boundaries, and the mechanism might be p53 activation by BANP, but it has not been fully elucidated 38. In addition, BANP could prevent tumour metastasis and invasion through the TGF-β pathway. TGF-β is a major protein in the regulation of tumour cell metastasis, invasion and epithelial-mesenchymal-transition (EMT). Overexpression of TGF-β1 has been associated with breast cancer, colon cancer, oesophageal cancer, hepatocellular carcinoma, etc., and it has been speculated that TGF-β1 is involved in mediating tumour progression, metastasis, angiogenesis and prognosis 39-40. In a study of stabilizing the cell genome width with BANP, BANP was found to downregulate TGF-β target genes such as mucin, fibre-adhesin, intercellular adhesion molecule (ICAM), cadherin 3, integrin α5 and junctional adhesion molecule (JAM2). The expression of these target genes increased the activity and metastasis of tumour cells 41-42. In this study, the results of analysis of the IntAct, Pathway Commons and STRING databases suggested that BANP could potentially interact with ZNF471. Through molecular biology experiments, we demonstrated not only the physical interaction between ZNF471 and BANP but also the promoting effect that ZNF471 had on BANP expression. In addition, the results of our cell biology functional experiments showed that BANP can inhibit the proliferation and metastasis of renal carcinoma cells, and induce apoptosis and cell cycle arrest in renal carcinoma cells.

Based on our findings, ZNF471 has great potential as a new diagnostic and therapeutic biomarker for RCC. On the one hand, the high expression level and CpG hypermethylation of ZNF471 in RCC are likely to be considered potential molecular markers in the future, which might assist pathologists in determining the clinicopathological characteristics of tumours and predicting prognosis. On the other hand, we could propose new methods using ZNF471 agonists, taking advantage of ZNF471's tumour suppressor role in RCC, for renal cancer therapy.

## Conclusion

In summary, ZNF471 could act as an important tumour suppressor in RCC and is often inactivated by promoter CpG hypermethylation. In addition, ZNF471 could interact with BANP and inhibit the activation of the PI3K/AKT/mTOR signalling pathway, thereby suppressing the malignant phenotype of RCC cells. The tumour-specific promoter methylation of ZNF471 might be a candidate diagnostic and therapeutic biomarker for RCC.

## Supplementary Material

Supplementary figures and tables.

## Figures and Tables

**Table 1:** The association between ZNF471 expression level in renal cell carcinoma and clinicopathological features of renal carcinoma patients (n=80)

	ZNF471 expression			
Characteristics	Low No. Cases	High No. Cases	Chi-squared test	P-value
All patients	(n=42)	(n=38)		
Gender			0.802	0.37
Male	19	21		
Female	23	17		
Age (years)			1.337	0.248
≤60	20	23		
>60	22	15		
Tumor stage			6.014	0.014
T1-T2	15	24		
T3-T4	27	14		
Tumor size			1.229	0.268
≤5cm	18	21		
>5cm	24	17		
Pathologic grade				
G1-G2	16	23	4.018	0.045
G3-G4	26	15		

**Table 2:** The association between ZNF471 methylation in renal cell carcinoma and clinicopathological features of renal carcinoma patients (n=80)

	ZNF471			
Characteristics	methylation	unmethylation	Chi-squared test	P-value
All patients	(n=58)	(n=22)		
Gender			0	1
Male	29	11		
Female	29	11		
Age (years)			0.348	0.555
≤60	30	13		
>60	28	9		
Tumor stage			4.586	0.032
T1-T2	24	15		
T3-T4	34	7		
Tumor size			0.018	0.894
≤5cm	27	12		
>5cm	21	10		
Pathologic grade			0.148	0.7
G1-G2	26	13		
G3-G4	22	9		

## Abbreviations

RCC: renal cell carcinoma
ZNF: zinc finger protein
C2H2-ZNF: C2H2 zinc finger protein
KRAB-ZFP: krüppel-associated box domain-containing zinc finger protein
DNMT: DNA methyltransferase
FAB: finger-associated box
FAR: finger-associated repeat
PVZ: pox virus and zinc finger
KRAB: krüppel-associated box
TCGA: the Cancer Genome Atlas
RT-PCR: reverse-transcription polymerase chain reaction
qRT-PCR: quantitative real-time polymerase chain reaction
PBS: phosphate-buffered saline
PI: propidium iodide
PMSF: phenylmethylsulfonyl fluoride
BCA: bicinchoninic acid
KEGG: Kyoto Encyclopedia of Genes and Genomes
Co‑IP: co‑immunoprecipitation
Aza: 5-Aza-2′-deoxycytidine
HE: hematoxylin and eosin
IHC: immunohistochemistry
TGF-β: transforming growth factor
EMT: epithelial interstitial transition
ICAM: intercellular adhesion molecule
