# Supplementary material for: ZNF471 Interacts with BANP to Reduce Tumour Malignancy by Inactivating PI3K/AKT/mTOR Signalling but is Frequently Silenced by Aberrant Promoter Methylation in Renal Cell Carcinoma
Source: Int J Biol Sci. 2024 Jan 1;20(2):643–63. doi: 10.7150/ijbs.89785 (PMC10758100; doi:10.7150/ijbs.89785)
Supplement: Supplementary file 1 — Supplementary figures and tables. [file ijbsv20p0643s1.pdf]

Supplementary Table 1 The general information of the patients in this study

Characteristics	All patients (n=80)
Gender	
Male	40
Female	40
Age（years）	
≤60	43
> 60	37
Tumor stage	
T1	19
T2	20
T3	21
T4	20
Tumor size	
≤5cm	39
> 5cm	41
Pathologic grade	
G1	18
G2	21
G3	23
G4	18

Supplementary Table 2 The primers used in this study

PCR	Primer	Sequence (5′-3′)
qRT-PCR	ZNF471-F	GAGATGACGAGTGAGATGAC
	ZNF471-R	TGACTTCCCATCTGCTTCTC
	GAPDH-F	CCAGCAAGAGCACAAGAGGAA
	GAPDH-R	GGTCTACATGGCAACTCAAGG
	E-cadherin-F	CGAGAGCTACACGTTCACGG
	E-cadherin-R	GGGTGTCGAGGGAAAAATAGG
	N-cadherin-F	TGCGGTACAGTGTAACTGGG
	N-cadherin-R	GAAACCGGGCTATCTGCTCG
	Vimentin-F	AGTCCACTGAGTACCGGAGAC
	Vimentin-R	CATTTCACGCATCTGGCGTTC
	Snail-F	ACTGCAACAAGGAATACCTCAG
	Snail-R	ACTGCAACAAGGAATACCTCAG
	BANP-F	GGCCATCCAGATTCAGTGAG
	BANP-R	AGCAGGACTCAAACGAAAGG
MSP	ZNF471-m44	TTTTGTTTTCGTTTTTTTCGTTC
	ZNF471-m6	ACGCGACTAAACCTTCGCG
	ZNF471-u44	GTTTTGTTTTTGTTTTTTTTGTTT
	ZNF471-u6	AAAAACACAACTAAACCTTCACA
BGS	ZNF471BGS1	GGTTTTTTTTATTTTTTTAGTAGTT
	ZNF471BGS4	TCCCACAACTCACTCCAATAA

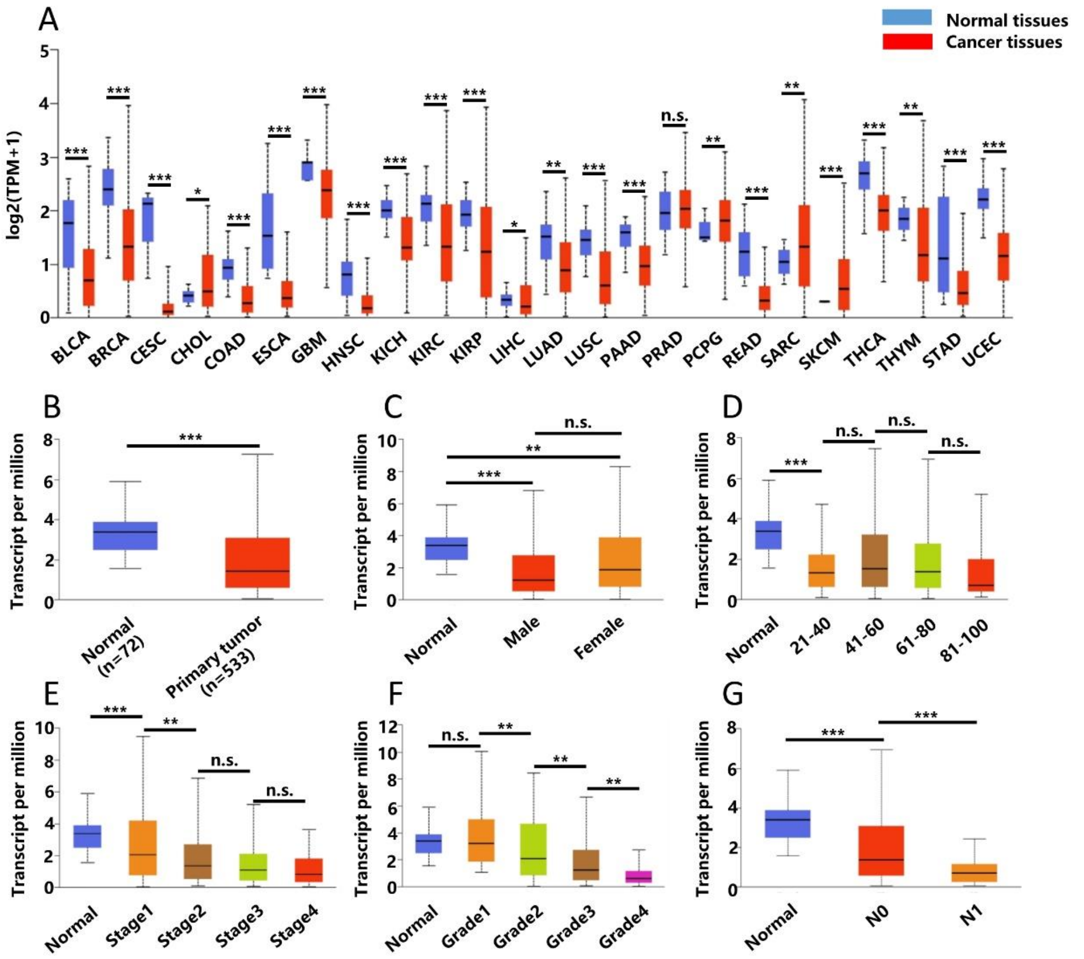

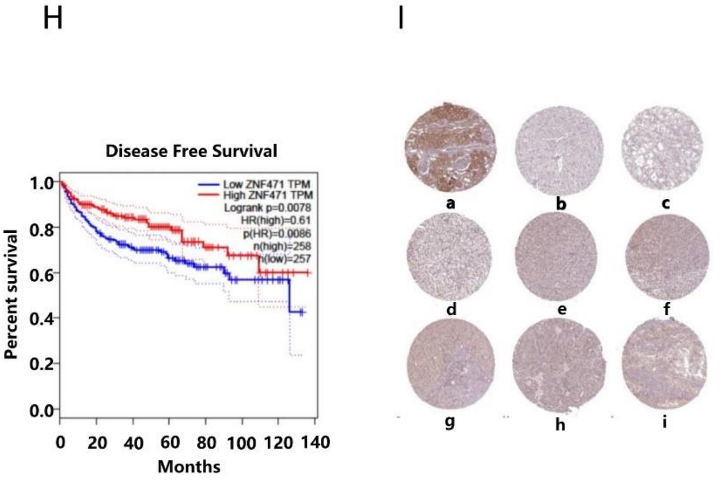

J

Tissue	Staining	Intensity
a	Medium	Moderate
b	Not detected	Negative
c	Not detected	Negative
d	Not detected	Negative
e	Low	Low
f	Low	Low
g	Low	Low
h	Low	Low
i	Low	Low

Supplementary Figure 1 The expression of ZNF471 in renal carcinoma. A. The expression of ZNF471 in most tumor tissues from The Cancer Genome Atlas (TCGA). B. The expression of ZNF471 in renal carcinoma from TCGA. C-G. The correlation between expression of ZNF471 and clinicopathologic feature of patients with renal carcinoma. H. The correlation between expression of ZNF471 and prognosis of patients with renal carcinoma. I-J. The protein expression level of ZNF471 in renal cancer tissues from The Human Protein Atlas. (n.s: $p > 0.05$, *$p < 0.05$, **$p < 0.01$,

***p < 0.001)

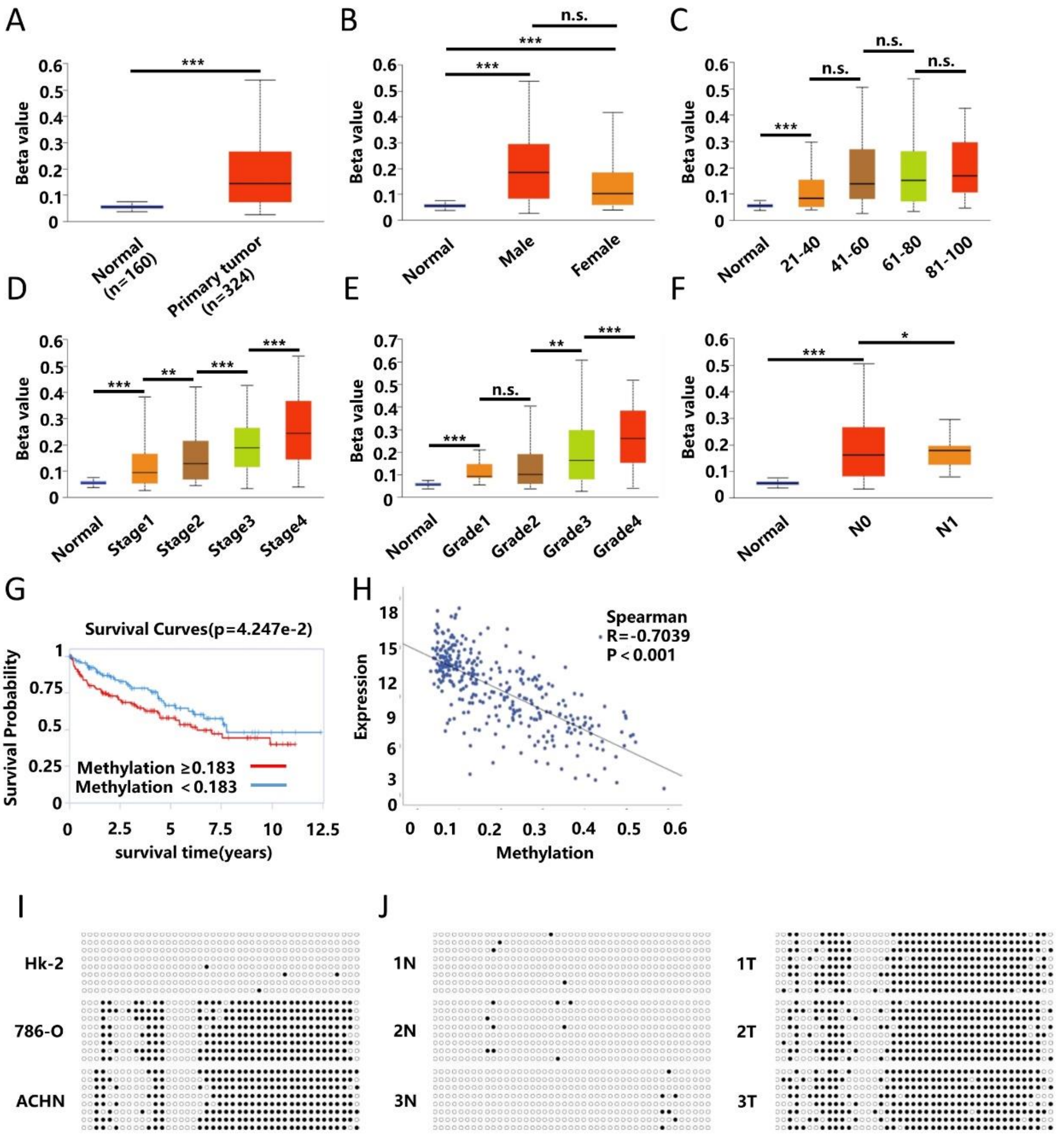

Supplementary Figure 2 The promoter methylation status of ZNF471 in renal carcinoma. A. The promoter methylation status of ZNF471 in renal carcinoma from The Cancer Genome Atlas (TCGA). B-F. The correlation between promoter methylation of ZNF471 and clinicopathologic feature of patients with renal carcinoma from TCGA. G. The correlation between promoter methylation of ZNF471 and prognosis of patients with renal carcinoma from EWAS Data Hub. H. The correlation between promoter methylation of ZNF471 and expression of ZNF471 in renal carcinoma from EWAS Data Hub. I. High-resolution methylation analysis of ZNF471 promoter by BGS in HK-2, 786-O and ACHN cells. J. High-resolution methylation analysis of ZNF471 promoter by BGS in

renal cancer tissues and adjacent normal tissues. (n.s: p > 0.05, *p < 0.05, **p < 0.01, ***p < 0.001)

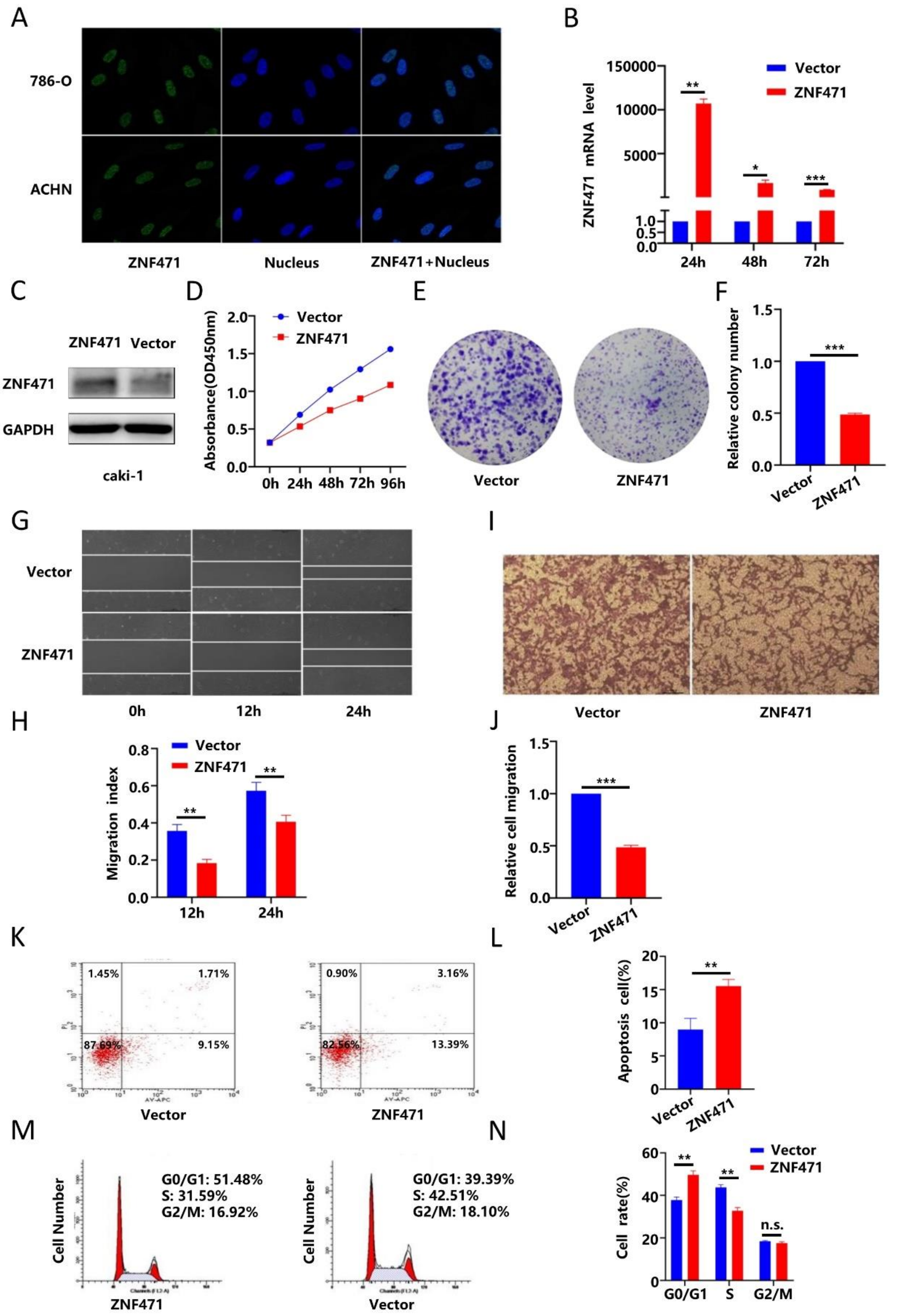

Supplementary Figure 3 The biological function of ZNF471 in renal cancer cell lines (caki-1). A. The subcellular localization of ZNF471 in 786-O and ACHN. B-C. The overexpression efficiency of ZNF471 in caki-1. D-F. The effect of ZNF471 on proliferation in caki-1. G-J. The effect of ZNF471 on invasion in caki-1. K-L. The effect of ZNF471 on apoptosis in caki-1. M-N. The effect

of ZNF471 on the cell cycle in caki-1. (n.s: p > 0.05, *p < 0.05, **p < 0.01, ***p < 0.001)

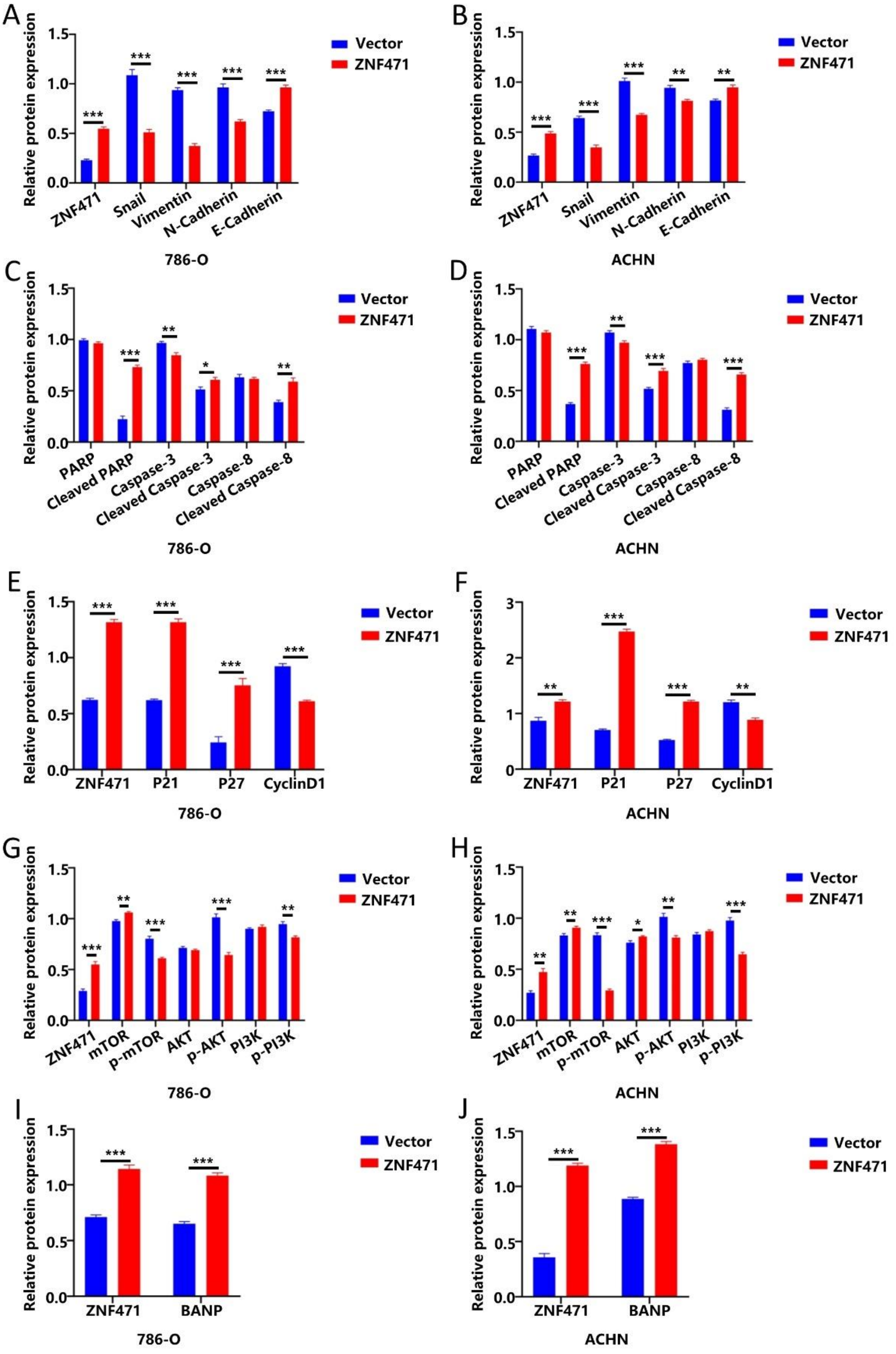

Supplementary Figure 4 A-B. The effect of ZNF471 on protein of E-cadherin, N-cadherin, vimentin and snail. C-D. The effect of ZNF471 on protein of caspase 3, cleaved caspase 3, caspase 8, cleaved caspase 8, PARP and cleaved PARP. E-F. The effect of ZNF471 on protein of p21, p27 and Cyclin D1. G-H. The effect of ZNF471 on key proteins of PI3K/AKT/mTOR pathways. I-J. The correlation

between the expression of ZNF471 and BANP. (n.s: $p > 0.05$, *$p < 0.05$, **$p < 0.01$, ***$p < 0.001$)
